# Understanding and Increasing Influenza Vaccination Acceptance: Insights from a 2016 National Survey of U.S. Adults

**DOI:** 10.3390/ijerph15040711

**Published:** 2018-04-10

**Authors:** Glen J. Nowak, Michael A. Cacciatore, María E. Len-Ríos

**Affiliations:** Center for Health and Risk Communication, Grady College of Journalism & Mass Communication, University of Georgia, 120 Hooper Street, Athens, GA 30602, USA; mcacciat@uga.edu (M.A.C.); lenriosm@uga.edu (M.E.L.-R.)

**Keywords:** influenza, influenza vaccination, adult vaccination, vaccine acceptance

## Abstract

*Background*: The percentage of adults in the U.S. getting seasonal influenza vaccination has not changed significantly since 2013 and remains far below the federal government’s 70% target. *Objective*: This study assessed and identified characteristics, experiences, and beliefs associated with influenza vaccination using a nationally representative survey of 1005 U.S. adults 19 years old and older. *Methods*: The sample was drawn from the National Opinion Research Center’s AmeriSpeak Panel, a probability-based panel designed to be representative of the U.S. household population. *Results*: Overall, 42.3% received an influenza vaccination in the past 12 months, with rates highest for non-Hispanic Whites and Blacks and those 65 years old and older. Hispanic respondents and those under 64 years old were much less likely to get an influenza vaccination. They were also less aware of the recommendation, less informed about influenza and the benefits of vaccination, and least confident in the vaccine. *Conclusions*: Increasing influenza vaccination coverage in the U.S. requires a greater focus on 19–64 year-olds, particularly those 50 to 64, Hispanics and continued focus on those with diabetes and asthma. Efforts need to increase awareness of influenza vaccination recommendations, foster a sense of being well informed about influenza vaccination benefits and the risks associated with non-vaccination, and increase confidence that there are meaningful benefits from receiving an influenza vaccination.

## 1. Introduction

Annual influenza vaccination has been recommended since 2010 for all U.S. adults by the country’s Advisory Committee on Immunization Practices (ACIP) [1], but little significant progress in increasing coverage has been made in recent years. The percentage of adults in the U.S. getting seasonal influenza vaccination has not significantly changed since 2013 and remains far below the 70% target established by the nation’s Healthy People 2020 program. Healthy People provides science-based, 10-year national objectives for improving the health of all Americans [2]. According to U.S. Centers for Disease Control and Prevention estimates (CDC), 42.2% of adults received an influenza vaccination in 2013–2014, 43.6% in 2014–2015, and 41.7% in 2015–2016 [3,4,5,6]. Early season coverage data suggested only about four of 10 U.S. adults would receive an influenza vaccination in 2016–2017 [3]. Even among U.S. adults with medical conditions (e.g., pulmonary disease, diabetes, asthma, or heart disease) influenza vaccination coverage is quite low, placing them at risk of influenza complications. While the recommendation has existed since the early 1960s, only about half of U.S. adults with high-risk conditions report getting a seasonal influenza vaccination [7,8]. 

Recent reviews provide insights into potential facilitators and barriers to influenza vaccination. Schmid et al. systematically reviewed 470 articles published between 2005 and 2016, involving a wide range of countries, and found sociodemographic variables, particularly gender and age, were the most reported yet the most inconsistent predictors of vaccination [9]. Stronger, more consistent relationships were found with level of vaccine confidence, influenza complacency, physician recommendation, past influenza illness, and vaccination experience. This is consistent with recent reviews involving both U.S. and European adults, which have generally found seasonal influenza vaccination associated with healthcare provider recommendations, perceptions of susceptibility to influenza and its severity, believing in influenza vaccine effectiveness, negative influenza experiences, and no negative seasonal influenza vaccination experiences [10,11,12]. Non-vaccination has generally been associated with the opposite [10,11,12]. Overall, surveys of U.S. adults, while primarily focused on 2009 H1N1 vaccination, have also found healthcare provider recommendations, susceptibility and severity perceptions, and past experience with influenza illness and vaccination correlated with seasonal influenza vaccination [13,14,15,16,17]. Racial and ethnic differences also have been frequently documented [18,19,20], with the 2015–2016 seasonal vaccination rates ranging from 44.5% for non-Hispanic Whites to 36.6% for non-Hispanic Blacks to 34.4% for Hispanics [4]. 

While previous research provides many helpful insights into acceptance and non-acceptance of seasonal influenza vaccination by U.S. adults, the findings generally emanate from review articles [9,10,11,12], surveys of adults at higher risk of influenza complications (e.g., older adults, pregnant women, and people with chronic health conditions) [7,11,21], or studies involving 2009 H1N1 vaccination [13,14,15]. This study built off this research by using a national probability survey of U.S. adults to assess: (1) influenza vaccination behavior and intentions; (2) knowledge, beliefs, and confidence regarding influenza illness and vaccination; and (3) self-reported experiences with influenza to identify characteristics, experiences, and beliefs associated with vaccination.

## 2. Materials and Methods 

### 2.1. Study Design and Sample

A general population sample of U.S. adults 19 years old and older was drawn from the National Opinion Research Center’s (NORC) AmeriSpeak Panel using sampling strata based on age, race/ethnicity, education, and gender. Funded and operated by NORC at the University of Chicago, AmeriSpeak is a probability-based panel designed to be representative of the U.S. household population [22,23]. In order to provide a nationally representative sample, AmeriSpeak uses the NORC National Sample Frame, which was constructed by NORC to cover over 99 percent of U.S. households. The 2016 AmeriSpeak Panel consisted of nationally representative housing units drawn from around 3 million households in the 2010 NORC National Sample Frame [22]. 

The survey was fielded in October 2016 in English, using Internet and phone modes. Prior to fielding the survey, a pretest was done with 25 participants to assess question wording and understanding. This resulted in a few additions to some of the question response categories. Approximately 86% of respondents completed the survey via the Internet, while 14% involved telephone interviews. Telephone interviews are used by NORC to strengthen the representativeness of the AmeriSpeak panel. Some people do not have Internet access and would be excluded from participating without the telephone option.

Panelists received the cash equivalent of $3 for completing the survey. The Grady College of Journalism & Mass Communication provided funding support for the survey. The University of Georgia Institutional Review Board reviewed the protocol and exempted the study (UGA STUDY00003509). Informed consent was obtained from all participants at the start of the survey regardless of whether they completed it using the Internet or through a telephone interview, with the consent process describing the study purpose, indicating it would take 15–18 min to complete, and informing them that no personal identifying information would be retained. Internet respondents were able to review and change their answers using a back button, while those interviewed by phone could change answers by telling the interviewer they wanted to make a change.

### 2.2. Survey Design and Measures

While both Internet and telephone were used in the data collection, a standardized questionnaire was used for the study. Health profile questions obtained self-reported current health status, when last visited a doctor for a routine health checkup, whether ever smoked and whether currently smoked, current health insurance status, and awareness of whether an annual influenza vaccine was recommended for them. Participants were also asked if they had ever been told by doctor or health professional that they had pre-diabetes, high blood sugar or diabetes, asthma, high cholesterol, or influenza/the flu, and if they were currently taking prescription drugs to treat their pre-diabetes, diabetes, cholesterol, or asthma.

Influenza vaccination was assessed by asking participants: (1) if they had received, planned to receive (probably or definitely), or planned not to receive (probably or definitely) a flu vaccination during the 2016–2017 season; (2) if they had received a flu shot in the arm or a nasal spray vaccination in past 12 months; and (3) if they had received the influenza vaccine in 2013, 2014 and/or 2015. Participants were asked if they had received a recommendation from a healthcare provider in the past year for a flu vaccination; and whether they ever delayed or declined a recommended vaccine for reasons other than illness or allergy and if so, which one(s).

Published qualitative research suggests past experience with influenza illness has or could motivate vaccination [10,11,12], but Schmid et al.’s 2017 comprehensive review suggests published surveys of U.S. adults have not reported using such measures [9]. Thus, in addition to having received an influenza diagnosis from a doctor, three additional measures of self-reported influenza experiences based on qualitative research [10,12] used here were “have you ever been sick with influenza—that is, flu which infected your lungs,” “how would you rate your last influenza illness (on a 1–5 scale, where ‘1’ was “not very severe” and ‘5’ was “very severe”), and “how many times would you estimate you have had influenza—or the flu-in your life?”

Items related to influenza illness and vaccine were based on findings from recent reviews and surveys as well as Thomson et al.’s 2016 “practical taxonomy” which organized determinants of vaccine uptake into five domains [24]. Four domains—access, affordability, awareness, and acceptance were assessed here; activation was not used because it involves strategies that are unlikely to be consciously recognized (i.e., “nudging”). Specific measures included: how well informed individuals believed they were regarding seasonal influenza, seasonal influenza vaccine, and seasonal influenza vaccine benefits and risks; how easy/difficult it would be to get an influenza vaccination if they wanted one; perceived likelihood of getting influenza; perceived seriousness of illness if they contracted influenza; perceived impact on daily life; likelihood of transmitting influenza to others; and confidence in the safety, effectiveness, and benefits of the influenza vaccine.

### 2.3. Statistical Analysis

All survey data were weighted by using the Current Population Survey of the U.S. Census to be representative of the U.S. population for age, gender, education, race/ethnicity, housing tenure, telephone status, and census division. Descriptive and multivariate analyses were calculated using SPSS version 24 (IBM, Armonk, NY, USA). The overall survey margin of error was +/−3.9%. Crosstabs were used to identify any associations between influenza vaccination behaviors and intentions, demographic characteristics, and influenza and flu vaccine belief and confidence measures. Hierarchical ordinary least squares (OLS) regression and binary logistic regression were used to control for variables that might simultaneously affect the dependent variables, thus offering a more stringent test of patterns. For all analyses, a *p*-value of ≤0.05 was considered significant.

## 3. Results

### 3.1. Response Rates and Study Sample

The overall response rate was 35.6% (1005 completed interviews from 2820 sampled units) with a 26.2% weighted household recruitment rate (RECR). As of November 2015, the average RECR for the AmeriSpeak panel was 36.9%, which compares favorably with rates of 3.5–8% found in other widely used U.S. national probability panels [23,25,26]. The median time to complete the survey was 19 min.

Comparisons of the respondents to U.S. population estimates found only two notable differences. Compared to U.S. census estimates, the final weighted sample had slightly more individuals from households with annual incomes of less than $30,000 (30.4% vs. 21.3%) and more non-married individuals (50.1% vs. 46.1%). It had fewer individuals from households with annual incomes greater than $125,000 (12.3% vs. 19.0%) and married (49.9% vs. 53.9%). Further, as shown in Table 1, while the overall percentage of Hispanic respondents was similar to the U.S. population, it under-represented Hispanics 55 years old and older.

All reported analyses are based on weighted data using a weight supplied by NORC. The NORC weight accounted for basic demographics, including age, sex, education, race/ethnicity, housing tenure, telephone status, and Census Division, as well as household and individual nonresponse [22,23].

Table 1 shows 64.5% of respondents were non-Hispanic Whites, 15.6% were Hispanic, and 11.7% were non-Hispanic Blacks. Fifty-four percent were 19–49 years old, 26.2% were 50–64 years old, and 19.8% were 65 years old or older. Hispanic respondents were more likely than White and Black respondents to be 19–44 years old, and less likely to be 55 years old and older (*p*’s ≤ 0.05), with only 2.6% of Hispanic respondents being 65 and older. Just under half of all respondents had health insurance through their employer, while 33.3% had Medicare (19.1%) or Medicaid (14.2%). Non-Hispanic Blacks were least likely to have health insurance through their employer (35.9%) and more likely to have Medicare or Medicaid (*p*’s ≤ 0.05). Most respondents (86.8%) characterized their health as “good” or better. About two-thirds had visited a doctor for a routine checkup in the past year, with this highest for those 65 and older (91.5%), 50–64 year-olds (76.4%), and non-Hispanic Blacks (80.9%), and lowest for Hispanics (54.7%) and 19–30 years old (45.5%) (*p*’s ≤ 0.05). Overall, between 17–20% of respondents reported having pre-diabetes, diabetes, or asthma, with pre-diabetes (36.6%) and diabetes (31.3%) rates much higher among non-Hispanic Blacks (*p*’s ≤ 0.05).

### 3.2. Influenza Vaccination Recommendation Knowledge

Table 1 also shows 73.5% of respondents indicated an annual influenza vaccine or shot would be recommended for them, but only 54.8% of Hispanic respondents were aware of the recommendation (*p* ≤ 0.05). Additional analyses, not included in Table 1, showed that the percentage who knew they should receive an annual influenza vaccination increased from 51.5% for 19–30 year-olds to 66.7% for 31–49 year-olds to 82.8% for 50–64 year-olds to 91.6% for those 65 years old and older (*p*’s ≤ 0.05). Additional analyses also indicated 83% of those with pre-diabetes and diabetes and 78.5% of those with asthma said an influenza vaccination would be recommended for them.

Overall, 56.1% of respondents had been told to receive an influenza vaccine by a healthcare provider in the past year, with the percentage lower among non-Hispanic Blacks (51.7%) and Hispanics (43.2%) as well as among 19–39 year-olds (39.2%) and 31–49 year-olds (49.4%) (*p*’s ≤ 0.05). Analyses not included in Table 1 indicated about 60% of those 50–64 and 78.4% of those 65 and older reported being told, as did 69% of those with pre-diabetes or diabetes and 66.5% of those with asthma. In addition, 61.8% of females reported being told versus 49.8% of males (*p* ≤ 0.05). Overall, among those who visited a doctor in the past year, 65.9% reported being told to get an influenza vaccination.

### 3.3. Influenza Vaccination Receipt and Intentions

Table 2 shows that 42.3% of respondents received an influenza vaccination in the past 12 months, with two-thirds doing so in October (40.8%) or September (27.7%). Only about 7% received an influenza vaccination in December or later. When asked about the current season (i.e., 2016–2017 for this survey), 13.5% indicated they had been vaccinated, while 40.5% said they definitely or probably would get vaccinated. One in four indicated they would definitely not get an influenza vaccination. When asked about 2013–2015, about one-third received a flu vaccination all three years, 20–25% received in one or two of those years, and 41.1% said none. Overall, 19.2% had delayed, and 29.5% had declined, getting an influenza vaccination for reasons other than illness or allergy.

Table 2 shows additional statistically significant differences with respect to influenza vaccination receipt. Influenza vaccination in past 12 months, for the 2016–2017 season, and receiving a vaccine each calendar year in 2013, 2014, and 2015 increased by age category. Vaccination receipt and intentions were highly positively correlated with being aware that one should receive an influenza vaccination (e.g., 58.7% of those aware received one in past 12 months versus 9.2% of unaware group) and being told by a physician or healthcare provider (63.9% vs. 16.0% for those not told) (*p*’s ≤ 0.05).

Additional analyses not included or shown in Table 2, examined associations with influenza vaccination receipt and intentions and pre-diabetes, diabetes, and asthma. Overall, receipt of an influenza vaccination in the past 12 months was higher for those with pre-diabetes (55.0%) or diabetes (56.5%) than those not reporting either condition (39%) (*p* ≤ 0.05). In addition, those with pre-diabetes or diabetes were more likely to have already received an influenza vaccination during the 2016–2017 season or to report an intention to get one (e.g., ~19% vs. 12% had already received) (*p*’s ≤ 0.05). With respect to asthma, no statistically significant differences were found in terms of getting a an influenza vaccination in the past 12 months or planning to get one in the survey year. However, only 31.4% of those with asthma said they had not received an influenza vaccination in any of the three previous years compared to 42.3% of all respondents (*p* ≤ 0.05). 

### 3.4. Influenza Vaccination Receipt and Intentions Based on Race/Ethnicity

Table 3 shows Hispanic respondents were much less likely to have received an influenza shot or vaccine in the past 12 months compared to non-Hispanic White, non-Hispanic Black, and those in “Other” racial/ethnic groups. Only 20.8% of Hispanics reported an influenza vaccination in past 12 months, and only 16.4% reported having received an influenza vaccination in each of the three previous calendar years. Just over half of the Hispanic respondents indicated they had not received an influenza vaccination in the previous three calendar years. With respect to the 2016–2017 influenza season, only 6.5% of Hispanic respondents had already received an influenza vaccination, with another 37% indicating they definitely or probably would receive one. Conversely, 19.3% of non-Hispanic Blacks and 14.7% of non-Hispanic Whites reported having already received an influenza vaccination during the 2016–2017 influenza season, with just under 40% also reporting having received flu vaccinations in each of the three previous calendar years.

With respect to timing of influenza vaccination, the only notable statistically significant difference involved more Hispanic respondents indicating they had received their last influenza vaccination in January/February (13.8% vs. 4.3% overall). There were no racial/ethnic differences with respect to having ever delayed a recommended influenza vaccination for reasons other than illness or allergy, but non-Hispanic Whites and Hispanics were more likely to have ever declined a recommended influenza vaccination for reasons other than illness or allergy than non-Hispanic Blacks (27.8% vs. 18.6%). 

### 3.5. Influenza Illness Beliefs and Self-Reported Experiences

Table 4 shows only 10.9% of respondents reported being “not well” or “not at all” informed about seasonal influenza, with the percentage of “well informed” increasing by age category. About two-thirds of those receiving an influenza vaccination in the past 12 months said they were “very well informed” about influenza. Additional analyses indicated that approximately 20% of non-Hispanic Blacks and Hispanics reported being not well or not at all informed about the risks associated with not getting an influenza vaccine (vs. 10% of non-Hispanic Whites) (*p*’s ≤ 0.05). 

Only about one-third perceived their likelihood of contracting influenza to be “very” or “somewhat” high if they were not vaccinated (35.7%), while 80.3% of those not vaccinated in the past 12 months characterized their likelihood of contracting influenza as “somewhat” or “very” low. Higher percentages, however, indicated having influenza would affect their daily life and that if they had the flu, they would be likely (41.7%) or very likely (31.8%) to pass it on to others. Age and getting an influenza vaccination were both related to stronger perceptions of susceptibility and severity.

Overall, 42.4% reported having been sick with influenza that infected their lungs, with 35% characterizing their last influenza illness as “not very severe” and 28.8% indicating it was severe or very severe. Having influenza was associated with flu vaccination in past 12 months, but severity of last illness was not. Most people self-reported having influenza 1–4 times in their lifetimes, with frequency increasing with age, but not associated with flu vaccination in past 12 months.

### 3.6. Beliefs Regarding Influenza Vaccine

Table 5 shows most were “somewhat” (37.2%) or “very well” (53%) informed regarding who should receive seasonal influenza vaccine, and this belief increased with age and among those who received the vaccine in 12 months prior. Similar results and patterns were found for those informed about seasonal influenza vaccine benefits (88.9% were “somewhat” or “very well informed) and the risks associated with not getting vaccinated (76.5% were “somewhat” or “very well informed”). 

Notably, 69% of those who had received an influenza vaccination in the past 12 months said they were “very well informed” about the benefits associated with getting seasonal influenza vaccine whereas 63% of those who did not said they were “somewhat,” “not well,” or “not at all” informed about the benefits (*p* ≤ 0.05). Nearly all believed getting an influenza vaccination would be easy to do should they want one, including the vast majority of those who did not get one in the past 12 months.

Table 5 also shows respondents with the highest levels of confidence in the influenza vaccine’s safety, effectiveness, and benefits were far more likely to have gotten an influenza vaccination in the past 12 months. Overall, 49.3% were “confident” or “very confident” in the vaccine’s safety; 43% in its benefits; and 40.6% in its effectiveness. Confidence levels for all three increased with age but were lower for those younger than 49 years old.

### 3.7. Regression Results

As Table 6 and Table 7 show, regression analyses examined the simultaneous impacts of the predictor variables on: influenza vaccination receipt in the past 12 months; influenza vaccination receipt or likelihood for the 2016–2017 flu season; influenza vaccination receipt in 2013, 2014, and 2015; and overall confidence in seasonal influenza vaccination as assessed by total score on a three item-index (i.e., confidence in safety, effectiveness, and benefits). Age and race/ethnicity were the most consistent demographic predictors of vaccination attitudes and behaviors, with older respondents more supportive of influenza vaccination and Hispanic respondents simultaneously more confident in the vaccination, but less likely to have received it in recent years or to report themselves likely to receive it during the upcoming flu season. Awareness of the recommendation and a recommendation from a physician were, overall, the strongest predictors of influenza vaccination receipt. Perceived likelihood of getting influenza, how well informed respondents believed themselves to be about influenza and influenza vaccination, and a three-item confidence index were also strong and frequent positive predictors of vaccination.

## 4. Discussion

### 4.1. Results Overview

In line with U.S. coverage surveys in 2013 to 2015 [4,5,6], about 4 in 10 U.S. adults received an annual influenza vaccination, with receipt significantly higher for those 65 years old and older and modestly higher for those with pre-diabetes or diabetes. It remained lower for young adults and Hispanics. To give a sense of context, according to data from the 2015–2016 Canadian Community Health Survey, 32.4% of Canadians 12 and older had gotten an influenza vaccination in the previous 12 months. Canadians aged 65 or older were nearly twice as likely to have received an influenza shot in the past year, compared with any other age group (60.8% for seniors, compared with less than 36% for all other age groups), with young adults aged 18 to 34 least likely to have received an influenza vaccination shot (19.6%) [28]. A 2017 European Centre for Disease Control (ECDC) technical report indicated that in the European Union, the aggregate vaccination rate was 41.8% with several countries influenza vaccination rates approaching 70% or more [28]. Unlike the U.S., the countries in the EU report did not officially recommend influenza vaccination for healthy adults 19 to 49 years old [29].

### 4.2. Influenza Vaccination Uptake

The results of this study not only indicate the U.S. remains far short of its goal of achieving 70% overall influenza vaccination coverage among those 18 years old and older, there has been little progress toward achieving that goal in recent years. Similar to the data reported by CDC, the Public Health Agency of Canada and the ECDC, this study found the highest influenza vaccination rates among those 65 years old and older [4,5,6,9,28,29]. Conversely, the results obtained here provide further evidence of the lack of progress, and the need for greater progress in fostering influenza vaccination among a number of sub-populations, especially those people 64 years old and younger and Hispanics. It was encouraging to find that respondents with pre-diabetes and diabetes were among those most likely to have received an influenza vaccination in the past 12 months. However, even among these groups, approximately 45% had not done so and the overall rate remains significantly below the 70% goal found in the Healthy People 2020 objectives. Further, one third of non-Hispanic Black respondents reported having pre-diabetes or diabetes, with only about half reporting receipt of a healthcare provider recommendation for an influenza vaccination in the past 12 months. Among those with asthma, uptake was comparable to the overall U.S. population rate (i.e., ~43%), which suggests more needs to be done to educate and persuade members of this sub-population.

### 4.3. Factors Related to Influenza Vaccination

As the regression analyses indicated, age and race/ethnicity were the most consistent demographic predictors for receipt of a flu vaccination in the past 12 months as well as intention to receive, or receipt, in the 2016–2017 influenza season. Age and race/ethnicity were also consistently associated with positive influenza vaccination beliefs and higher levels of confidence in influenza vaccination safety, effectiveness, and benefits. Hispanic respondents, who were primarily 19 to 44 years old, had the lowest levels of awareness regarding the universal influenza vaccination recommendation, were least likely to have been told in the past year by a healthcare provider that they should receive an influenza vaccination, and were most likely to have declined a recommended influenza vaccination.

In the case of Hispanics, our data corroborate previous findings illustrating a gap in influenza vaccination for this sub-population in the U.S. [19] As the results obtained here suggest, one reason for the gap is that Hispanics (54.7%) were less likely than non-Hispanic Black respondents (80.9%) and non-Hispanic Whites (69.6%) to have seen a physician in the past year for a routine check-up. Since healthcare provider recommendations are a significant impetus for individuals getting an influenza vaccination, it would be expected that fewer encounters with healthcare providers, especially during key influenza vaccination months, would impede vaccination [30,31,32]. Moreover, the results obtained here suggest only about half of the Hispanic respondents who visited a healthcare provider in the past year were told they should receive an influenza vaccination. Given that at least one third of Hispanic respondents reported having pre-diabetes, diabetes, or asthma, this is a worrisome finding. It suggests missed opportunities to protect people at risk of serious complications of flu infections [8,33].

While the results also showed recommendation awareness mattered, they suggest that believing one is “very well informed” with respect to who should receive an influenza vaccine, the benefits of vaccination, and the risks associated with not being vaccinated matters more. Overall, 75% of the adults were aware they should receive an influenza vaccination, but only about half of the respondents considered themselves “very well informed” across the three measures used. For each measure, those who indicated that they were “well informed” were nearly four times more likely to have received an influenza vaccination in the past 12 months than those who said they were “not well informed” or “not at all” informed. Levels of recommendation awareness and being informed were correlated with age, race/ethnicity, and gender. Those 65 years old, non-Hispanic White or non-Hispanic Black, and female had the highest levels of awareness and perception that they were “well informed.” Hispanics, 19–49 year-olds, and males were most likely to be the least aware and informed. Notably, 50–64 year-olds were more similar in awareness and being informed to 31–49 year-olds than those 65 and older, suggesting they are not being well reached with influenza-related information and/or they pay little attention to such information. Knowing where to obtain an influenza vaccination was not a factor as almost all respondents knew where to obtain a flu vaccination.

The findings also reaffirmed the importance of a healthcare provider recommendation [9,12,33,34,35]. Two-thirds of those who received a provider recommendation got an influenza vaccination in the past 12 months, whereas 84% of those not getting a recommendation did not. Confidence in influenza vaccine safety, effectiveness, and personal benefit was also higher among those who received a provider recommendation (e.g., 53–61% having high confidence levels vs. 25.1–35.2%, *p*’s ≤ 0.05). However, while older adults and females were very likely to have seen a physician in the past year for a routine medical exam and to receive an influenza vaccination recommendation that was not usually the case for respondents who were Hispanic, male, or 49 years older and younger.

This study also obtained findings in two relatively unexamined domains—influenza vaccination-related confidence and self-reported experiences with influenza illness. With respect to confidence, the results suggest that influenza vaccination-related confidence matters. Individuals who had the highest levels of confidence in influenza vaccination safety, effectiveness, and benefits were seven times more likely to have received an influenza vaccination in the past 12 months than those who expressed little confidence. Overall, however, less than half of respondents reported having high levels of confidence in influenza vaccine effectiveness and benefits, with most of those being people 65 years old and older.

With respect to influenza illness experience, the results obtained here indicated a relatively small association with influenza vaccination. Across all age groups, just over 40% reported having had influenza sometime in their life. Reporting having had influenza was associated with vaccination, but self-reported severity and frequency of flu illness were not. Surprisingly, those who indicated their last influenza illness was quite severe were not found to have a higher likelihood of receiving a flu vaccination. Respondents who were 50–64 years old were most likely to characterize their last bout of flu as relatively severe, but this was not associated with higher vaccination even among this group. That said, having a somewhat or severe flu illness was associated with higher confidence that one would benefit from vaccination.

### 4.4. Implications for Efforts to Increase Influenza Vaccination

The findings obtained here have a number of implications for increasing U.S. influenza vaccination rates, with potential relevance for other countries. First, the findings illustrate the challenges and needs related to adults’ awareness and knowledge of influenza and influenza vaccination recommendations. The results here indicate that six years after the U.S. CDC expanded the influenza vaccination recommendation to encompass all adults 18 years and older [1], approximately 75% of the adults surveyed here were aware they should receive a seasonal influenza vaccine. That is significant progress in a relatively short period of time, but also indicates one in four American adults remain unaware of the recommendation. More importantly, the results obtained here indicate (1) larger percentages of 19–50 year-old adults as well as many Hispanic Americans are unaware and (2) many of those who are aware of the recommendation do not perceive themselves to be very well informed regarding who should receive the influenza vaccine, the benefits associated with getting the vaccine, and the risks associated with not receiving it. Both more and better influenza vaccination-related communication and education efforts are needed, with it likely being that targeted and tailored messages and materials will need to be used [9,10,12,35,36,37].

Second, while the findings reaffirm the importance of having and fostering healthcare provider recommendations, they also highlight the need to find ways to reach adults in non-healthcare settings, including with messages from healthcare providers. It is unrealistic to expect that more visits to healthcare providers is an effective or efficient approach to fostering influenza vaccination, including because not all times of the year are helpful when it comes to influenza vaccination (e.g., in the U.S., September through January are typically the most relevant months). Given that large percentages of 19–44 year-olds, including Hispanic Americans, may not have a healthcare provider visit in a typical year, it is important to find other ways to reach them with information about influenza and influenza vaccination. This will necessitate research to identify the media Hispanics rely on for health and medical information. It is also likely the case that with respect to Hispanic Americans, efforts are needed to improve physician-patient interactions [38], including by addressing health literacy factors and improving cultural competencies [39,40].

Third, the findings suggest that more efforts are needed in the U.S. to persuade those 50 to 64 years old to get an annual influenza vaccination. While it is somewhat expected that influenza vaccination rates would be lower among 19 to 49 year-olds, particularly among those without a chronic health condition, this study found only about half of those 50–64 years old received an influenza vaccination in the past 12 month and only about a third appeared to have made a habit of receiving a seasonal influenza vaccination (i.e., had received one in each of the three previous years). Further, the findings here suggest that while improvement is needed in getting more healthcare providers to provide their 50 to 64 year-old patients with a recommendation, more than that will likely be needed to achieve significant success [12,36,37]. For instance, 82.8% of the 50–64 year-olds surveyed here said they were aware an influenza vaccination was recommended for people their age, and 41% characterized their last influenza illness as quite severe. However, not only did just 51.5% report getting an influenza vaccination in the past 12 months, 29.9% reported having declined a recommended influenza vaccination for reasons other than illness or allergy. This suggests that even though 50 to 64 year-olds are approaching an age category where vulnerability to influenza complications increases (i.e., those 65 years old and older), a large percentage have benefit-risk perceptions or calculations that are worrisome. At a minimum, communication and education efforts must find ways to get those who forego influenza vaccination to believe the benefits are greater than the risks [10,35,36]. It may also be the case that a significantly improved influenza vaccination will be needed to achieve such a change in perceptions [12].

A final implication of these findings is that many adults appear to consider influenza to be either an unlikely health threat or one that would be “manageable” (i.e., would not cause serious illness). This is in line with recent reviews [9,10,11,12], which have found that perceptions regarding susceptibility and severity of influenza were highly related to vaccination. In general, most influenza vaccine acceptors believe they have a high likelihood of contracting influenza, that the resulting illness will be somewhat or very serious and having influenza would greatly affect their daily life. Conversely, non-vaccinators often perceive less susceptibility to influenza, consider influenza not a serious illness, and believe being ill would have little to modest impact on their daily lives. To the extent these beliefs have been shaped by influenza and vaccine experiences to date, achieving behavior change with information alone will be difficult [10,12,36,37,40]. That said, in this study, most non-vaccinators considered themselves somewhat or not well informed about influenza and the influenza vaccine, including many not realizing they could transmit their influenza infection to others. In addition, given many 50–64 year-olds reported having had a severe case of influenza, efforts that highlight the ability of influenza vaccination to reduce the possibility of severe influenza illness may bolster their confidence that they would benefit from vaccination.

## 5. Limitations

This study has notable limitations. First, as with most studies that involve surveys, the findings are based on self-reported data. Influenza immunization was not validated with medical records, and validity studies have shown that parental report (for children) and self-report (for adults) does overestimate influenza vaccination coverage [41,42,43]. The influenza vaccination rates obtained here, however, are in line with those found by the U.S. CDC in the 2013 to 2016 timeframe [3,4,5,6]. In addition, the U.S. CDC has employed similar survey research methods (i.e., the use of nationally representative, probability-based panels, including those established by NORC) in achieving similar findings related to U.S. adults’ seasonal influenza vaccination [3,44]. Second, it is also the case that response rates for surveys that involve random probability sampling are much lower compared to an era where landline telephones were dominant. As cellphones have replaced landlines, survey non-response rates have greatly increased [25,26]. As a result, survey research in the U.S. now mostly involves the use of large nationally representative panels, such as the one used here [25]. While this typically results in response rates similar to those reported here, it is also the case these panels are designed to achieve representativeness and generalizability [22,23,25]. Third, as this study illustrates, there are some groups that remain underrepresented in these panels. In this case, there were relatively few Hispanic respondents 65 years old and older. Some of the findings related to Hispanic respondents would likely be different if more older Hispanics had been surveyed. Fourth, this study used a cross-sectional survey and as such, the findings can only be suggestive of causal relationships; surveys such as these are not able to test or confirm causal relationships. Finally, as Schmid et al.’s 2017 comprehensive review illustrated, studies have found that in addition to the variables examined here, a number of determinants can influence adults’ acceptance of influenza vaccination [9].

## 6. Conclusions

This study updates the current state of adult influenza vaccination acceptance in the U.S. and identifies challenges and opportunities. The findings indicate progress remains slow and they highlight the need for efforts that especially focus on 50–64 year-olds, Hispanics, and non-Hispanic Blacks who have pre-diabetes or diabetes, and continued efforts directed at those with diabetes or asthma. Efforts to encourage influenza vaccination need to increase awareness, generate a sense of being “very well informed” about the benefits of influenza vaccination and the risks associated with not being immunized, and instill confidence that one benefits from vaccination. Those efforts, however, need to recognize many non-vaccinators believe influenza, for themselves, to not be a significant health threat or illness. For these individuals, healthcare providers and influenza vaccination efforts should place emphasis on the vaccine’s ability to reduce the likelihood of severe influenza illness and transmission of influenza to others, who may suffer severe consequences. More also needs to be done to assist and encourage immunization programs and healthcare providers in reaching those with health conditions that elevate risk for influenza complications, including non-Hispanic Blacks with pre-diabetes or diabetes.

## Figures and Tables

**Table 1:** Respondent demographics and health profile.

	Overall	Non-Hispanic White	Non-Hispanic Black	Hispanic	Other
*N*	*1005*	*649*	*118*	*157*	*81*
**Characteristic**					
**Gender**					
Male	48.2%	48.7%	41.3%	48.6%	52.9%
Female	51.8%	51.3%	58.7%	51.4%	47.1%
**Age**					
19–24	10.6%	8.6% ^a^	9.3% ^a^	22.2% ^b^	4.9% ^a^
25–34	19.3%	14.9% ^a^	19.5% ^a,b^	33.5% ^c^	27.2% ^b,c^
35–44	14.7%	13.6% ^a^	11.0% ^a^	20.9% ^b^	17.3% ^a,b^
45–54	18.3%	19.9% ^a^	15.3% ^a,b^	10.8% ^b^	25.9% ^a^
55–64	17.2%	18.3% ^a,b^	24.6% ^b^	10.1% ^c^	11.1% ^a,c^
65–74	11.9%	15.1% ^a^	12.7% ^a,b^	1.3% ^c^	6.2% ^b^
75 and older	7.9%	9.6% ^a^	7.6% ^a^	1.3% ^b^	7.4% ^a^
**Education**					
Less than high school	10.6%	8.0% ^a^	19.5% ^b^	16.6% ^b^	6.3% ^a^
High school	29.1%	29.6%	28.8%	31.2%	22.5%
Some college	21.4%	21.3%	18.6%	26.1%	16.3%
College graduate	28.2%	30.8% ^a^	19.5% ^b^	21.7% ^b^	33.8% ^a^
Graduate education	10.7%	10.3% ^a^	13.6% ^a,b^	4.5% ^c^	21.3% ^b^
**Employment status**					
Working as employee	47.0%	46.4%	43.2%	54.1%	42.7%
Self-employed	9.3%	9.4% ^a,b^	5.1% ^b^	8.9% ^a,b^	15.9% ^a^
Not working	23.1%	19.6% ^a^	32.2% ^b^	33.1% ^b^	19.5% ^a^
Retired	20.6%	24.7% ^a^	19.5% ^a^	3.8% ^b^	22.0% ^a^
**Annual household income**					
$49,999 or under	51.4%	46.1% ^a^	69.5% ^b^	61.1% ^b,c^	48.1% ^a,c^
$50,000 or over	48.6%	53.9% ^a^	30.5% ^b^	38.9% ^b,c^	51.9% ^a,c^
**Health/medical insurance coverage**					
Insurance through employer	44.8%	45.4% ^a^	35.9% ^b^	49.0% ^a^	44.2% ^a,b^
Insurance purchased directly	8.0%	7.2% ^a^	5.1% ^a^	7.8% ^a^	19.5% ^b^
Medicare	19.1%	21.8% ^a^	27.4% ^a^	6.5% ^b^	9.1% ^b^
Medicaid or related type	14.2%	10.6% ^a^	20.5% ^b,c^	26.1% ^c^	10.4% ^a,b^
Other, including Veterans Administration	14.0%	15.0%	11.1%	10.5%	16.9%
**Self-reported general health status**					
Excellent	13.0%	13.0%	11.0%	13.5%	16.0%
Very good	37.5%	39.2%	32.2%	37.8%	30.9%
Good	36.3%	35.5%	39.0%	37.2%	38.3%
Fair	10.5%	9.3% ^a^	16.9% ^b^	9.6% ^a,b^	12.3% ^a,b^
Poor	2.6%	3.1%	0.8%	1.9%	2.5%
**Last visited a doctor for a routine checkup**					
Within the past year	68.0%	69.6% ^a^	80.9% ^b^	54.7% ^c^	63.2% ^a,c^
1 year but less than 2 years ago	14.0%	13.0% ^a^	10.4% ^a^	20.7% ^b^	14.5% ^a,b^
2 years but less than 4 years ago	7.4%	7.1%	6.1%	10.0%	6.6%
4 or more years ago	9.4%	9.4% ^a^	2.6% ^b^	12.7% ^a^	13.2% ^a^
Never	1.1%	0.9%	0.0%	2.0%	2.6%
**Ever smoked at least 100 cigarettes in life?**					
Yes	42.4%	48.4% ^a^	30.2% ^b^	28.6% ^b^	36.3% ^b^
No	57.6%	51.6% ^a^	69.8% ^b^	71.4% ^b^	63.8% ^b^
**Do you now smoke cigarettes?**					
Every day	27.8%	28.5%	34.3%	19.0%	23.3%
Some days	12.1%	8.3% ^a^	40.0% ^b^	23.8% ^b,c^	6.7% ^a,c^
Not at all	60.1%	63.1% ^a^	25.7% ^b^	57.1% ^a^	70.0% ^a^
**Have you ever been told by a doctor that you have or had … (“Yes” responses)**					
Pre-diabetes or borderline diabetes	20.9%	20.0% ^a^	36.6% ^b^	16.4% ^a^	13.9% ^a^
High blood sugar or diabetes	17.3%	16.3% ^a^	31.3% ^b^	10.7% ^a^	17.5% ^a^
Asthma	17.0%	17.0%	16.2%	15.9%	20.8%
High cholesterol	33.8%	35.8% ^a^	42.6% ^a^	16.4% ^b^	39.5% ^a^
Influenza or the flu	46.5%	53.0% ^a^	31.8% ^b^	34.7% ^b^	39.5% ^b^
**Are you currently taking prescription drug to treat … (“Yes” responses)**					
Pre-diabetes or borderline diabetes	9.3%	7.5% ^a^	23.3% ^b^	7.1% ^a^	7.7% ^a^
High blood sugar or diabetes	10.8%	9.2% ^a^	25.0% ^b^	7.4% ^a^	8.9% ^a^
Asthma	8.8%	9.6%	8.6%	5.2%	11.4%
High cholesterol	22.6%	23.5% ^a^	33.0% ^b^	9.7% ^c^	25.3% ^a,b^
**In the past year, has a physician or healthcare provider told you that you should receive an influenza or flu vaccine?**					
Yes	56.1%	60.6% ^a^	51.7% ^a,b^	43.2% ^b^	51.3% ^a,b^
No	43.9%	39.4% ^a^	48.3% ^a,b^	56.8% ^b^	48.8% ^a,b^
**Is an annual flu vaccine or shot something that would be recommended for you? That is, should someone your age receive—or have received—an annual flu vaccine or shot?**					
Yes	73.5%	77.9% ^a^	71.8% ^a^	54.8% ^b^	73.6% ^a^
No	26.5%	22.1% ^a^	28.2% ^a^	45.2% ^b^	26.4% ^a^

Note: Comparisons are made across columns by row using the Bonferroni adjusted *p*-value approach with an alpha value of ≤0.05. All possible pairwise comparisons are made between non-Hispanic Whites, non-Hispanic Blacks, Hispanics, and Others. Rows with no superscripts have no significant differences. Within rows that do have superscripts, any two values that share a superscript are not significantly different from one another; values that do not have a common superscript are significantly different (e.g., a, b, and c are significantly different from one another). For example, in the table there are no significant differences in gender distribution across the different race/ethnicity categories; however, there are several significant differences in the percentage of 25–34 year-olds based on race/ethnicity.

**Table 2:** Influenza vaccination experience and intentions based on age, awareness of recommendation, and whether they have been advised by a physician to get a flu vaccine.

	Over-All (%)	Age (%)				Aware of Recommendation (%)		Told by Physician to Get Flu Vaccine (%)	
		19–30	31–49	50–64	65+	Yes	No	Yes	No
*N*	*1005*	*212*	*330*	*264*	*199*	*645*	*233*	*549*	*430*
**During past 12 months, have you received a flu shot or vaccine?**									
Yes	42.3	20.5 ^a^	33.7 ^b^	51.5 ^c^	66.7 ^d^	58.7 ^a^	9.2 ^b^	63.9 ^a^	16.0 ^b^
No	57.7	79.5 ^a^	66.3 ^b^	48.5 ^c^	33.3 ^d^	41.3 ^a^	90.8 ^b^	36.1 ^a^	84.0 ^b^
**If yes, in which month did you receive your most recent flu vaccine?**									
September	27.7	39.0	23.9	24.4	30.5	28.2	27.8	29.0	22.4
October	40.8	14.6 ^a^	40.4 ^b^	44.9 ^b^	45.0 ^b^	42.2 ^a^	11.1 ^b^	42.0	32.8
November	18.2	31.7 ^a^	19.3 ^a,b^	17.3 ^b^	14.5 ^b^	16.9 ^a^	38.9 ^b^	16.6	25.4
December	2.6	2.4 ^a,b^	0.0 ^b^	1.6 ^a,b^	6.1 ^a^	3.0	0.0	2.4	4.5
January/February	4.3	9.8 ^a^	5.5% ^a^	5.5 ^a^	1.5 ^b^	3.2 ^a^	11.1 ^b^	3.9	7.5
**How likely are you to get a flu Vaccination this season (2016–2017)—that is, this Fall or Winter?**									
Already received	13.5	5.2 ^a^	9.5 ^a,b^	11.7 ^b^	31.2 ^c^	18.6 ^a^	3.4 ^b^	21.2 ^a^	4.4 ^b^
Will definitely get one	26.7	14.2 ^a^	21.0 ^b^	33.7 ^c^	40.2 ^c^	38.0 ^a^	6.4 ^b^	38.0 ^a^	12.4 ^b^
Will probably get one	13.8	18.5 ^a^	13.4 ^a,b^	14.8 ^a^	8.0 ^b^	16.4 ^a^	8.6 ^b^	13.9	13.8
Will probably not get one	18.9	27.0 ^a^	19.8 ^a,b^	19.3 ^b^	8.5 ^c^	12.2 ^a^	25.3 ^b^	10.8 ^a^	28.0 ^b^
Will definitely not get one	27.0	35.1 ^a^	36.3 ^a^	20.5 ^b^	12.1 ^c^	14.7 ^a^	56.2 ^b^	16.2 ^a^	41.5 ^b^
**Thinking about 2013, 2014, and 2015, in how many of those years did you get a flu vaccination?**									
Received in all three years	35.7	15.8 ^a^	25.3 ^b^	35.4 ^c^	72.1 ^d^	51.0 ^a^	5.0 ^b^	53.7 ^a^	12.9 ^b^
Received in two years	10.7	12.6 ^a,b^	9.6 ^b^	16.0 ^a^	4.1 ^c^	12.9 ^a^	5.4 ^b^	12.1	9.0
Received one year	12.5	17.9 ^a^	12.7 ^a,b^	12.2 ^a,b^	7.6 ^b^	11.1	15.8	11.2	12.7
None	41.1	53.7 ^a^	52.5 ^a^	36.5 ^b^	16.2 ^c^	25.0 ^a^	73.9 ^b^	23.0 ^a^	65.4 ^b^
**Have you ever delayed getting a recommended flu vaccination for reasons other than illness or allergy?**									
Yes	19.2	15.6 ^a^	16.1 ^a^	23.4 ^b^	22.7 ^a,b^	21.9 ^a^	15.0 ^b^	20.6	17.0
**Have you ever declined getting a recommended flu vaccination for reasons other than illness or allergy?**									
Yes	29.5	28.3 ^a^	36.7 ^b^	29.9 ^a,b^	18.1 ^c^	22.9 ^a^	45.3 ^b^	24.8 ^a^	36.3 ^b^

Note: Comparisons are made across columns by row using the Bonferroni adjusted *p*-value approach with an alpha value of ≤0.05. All possible pairwise comparisons are made between categories of a given variable (e.g., age). For any given variable, rows with no superscripts have no significant differences. Within rows that do have superscripts, any two values that share a superscript are not significantly different from one another; values that do not have a common superscript are significantly different (e.g., a, b, and c are significantly different from one another). For example, in the table there are no significant differences based on age in the percentage of respondents who reported getting a flu vaccine in September, but there are significant differences based on age in the percentage of respondents who reported getting a flu vaccine in October, with the youngest group of respondents significantly less likely than the other age groups to report having received a flu vaccine in that month.

**Table 3:** Influenza vaccination experience and Intentions based on race/ethnicity.

	Overall (%)	Non-Hispanic White (%)	Non-Hispanic Black (%)	Hispanic (%)	Other (%)
*N*	*1005*	*649*	*118*	*157*	*81*
**During past 12 months, have you received a flu shot or vaccine?**					
Yes	42.3	45.4 ^a^	50.9 ^a^	20.8 ^b^	44.4 ^a^
No	57.7	54.6 ^a^	49.1 ^a^	79.2 ^b^	55.6 ^a^
**If yes, in which month did you receive your most recent flu vaccine?**					
September	27.7	27.6	33.3	27.6	20.0
October	40.8	43.8	33.3	27.6	40.0
November	18.2	16.9	14.8	27.6	28.6
December	2.6	3.4	1.9	0.0	0.0
January/February	4.3	3.1 ^a^	5.6 ^a^	13.8 ^b^	5.8 ^a^
**How likely are you to get a flu Vaccination this season (2016–2017)—that is, this Fall or Winter?**					
Already received	13.5	14.7 ^a,b^	19.3 ^b^	6.5 ^c^	8.8 ^a,c^
Will definitely get one	26.7	27.6 ^a^	31.1 ^a^	18.2 ^b^	27.5 ^a,b^
Will probably get one	13.8	12.0 ^a^	10.1 ^a^	18.8 ^b^	25.0 ^b^
Will probably not get one	18.9	20.8 ^a^	15.1 ^a,b^	19.5 ^a^	8.8 ^b^
Will definitely not get one	27.0	24.8 ^a^	24.4 ^a^	37.0 ^b^	30.0 ^a,b^
**Thinking about 2013, 2014, and 2015, in how many of those years did you get a flu vaccination?**					
Received in all three years	35.7	39.7 ^a^	37.9 ^a^	16.4 ^b^	33.8 ^a^
Received in two years	10.7	9.5	13.8	12.9	12.5
Received one year	12.5	11.0 ^a^	12.1 ^a,b^	19.3 ^b^	15.0 ^a,b^
None	41.1	39.7 ^a^	36.2 ^a^	51.4 ^b^	38.8 ^a,b^
**Have you ever delayed getting a recommended flu vaccination for reasons other than illness or allergy?**					
Yes	19.2	19.8	19.5	17.2	17.3
**Have you ever declined getting a recommended flu vaccination for reasons other than illness or allergy?**					
Yes	29.5	31.9 ^a^	18.6 ^b^	27.8 ^a,b^	29.6 ^a,b^

Note: Comparisons are made across columns by row using the Bonferroni adjusted *p*-value approach with an alpha value of ≤0.05. All possible pairwise comparisons are made between non-Hispanic Whites, non-Hispanic Blacks, Hispanics, and Others. Rows with no superscripts have no significant differences. Within rows that do have superscripts, any two values that share a superscript are not significantly different from one another; values that do not have a common superscript are significantly different (e.g., a, b, and c are significantly different from one another). For example, in the first row of results, non-Hispanic Whites (45.4%) do not differ significantly from non-Hispanic Blacks (50.9%) in terms of whether they had received a flu shot or vaccine during the past 12 months because those values share the same superscript of ‘a’. However, Hispanics (20.8%) differ significantly from all of the other groups because they are the only group with the superscript ‘b’.

**Table 4:** Influenza illness beliefs and experience.

	Overall	Age				Got Flu Vaccine in Last 12 Months	
		19–30	31–49	50–64	65+	Yes	No
*N*	*1005*	*212*	*330*	*264*	*199*	*418*	*571*
**How well informed would you say you are regarding seasonal influenza or flu?**							
Very well informed	46.2%	28.0% ^a^	47.0% ^b^	47.5% ^b^	60.4% ^c^	63.5% ^a^	33.5% ^b^
Somewhat informed	42.9%	51.1% ^a^	42.7% ^a,b^	43.6% ^a,b^	35.0% ^b^	34.1% ^a^	49.5% ^b^
Not well informed	8.0%	17.2% ^a^	7.2% ^b^	6.9% ^b^	1.5% ^c^	1.2% ^a^	12.7% ^b^
Not at all informed	2.9%	3.8%	3.1%	1.9%	3.0%	1.2% ^a^	4.2% ^b^
**In general, if you do not get a flu vaccination, what do you think your chances are of getting the flu?**							
Very high	9.8%	2.9% ^a^	9.4% ^b^	10.8% ^b,c^	15.7% ^c^	15.6% ^a^	5.2% ^b^
Somewhat high	25.9%	14.0% ^a^	24.9% ^b^	27.8% ^b,c^	35.7% ^c^	41.7% ^a^	14.5% ^b^
Somewhat low	32.7%	42.1% ^a^	34.0% ^a,b^	30.7% ^b,c^	24.9% ^c^	26.9% ^a^	37.2% ^b^
Very low	31.6%	40.9% ^a^	31.6% ^b^	30.7% ^b^	23.8% ^b^	15.8% ^a^	43.1% ^b^
**In general, if you get the flu, how serious do you think the illness would be for you personally?**							
Very serious	11.9%	3.8% ^a^	12.6% ^b^	13.1% ^b^	17.8% ^b^	16.6% ^a^	8.6% ^b^
Somewhat serious	32.7%	26.1% ^a^	24.5% ^a^	40.4% ^b^	43.1% ^b^	43.1% ^a^	24.8% ^b^
Not very serious	46.7%	56.9% ^a^	54.0% ^a^	38.8% ^b^	34.0% ^b^	35.9% ^a^	54.8% ^b^
Not at all serious	8.7%	13.3% ^a^	8.9% ^a,b^	7.7% ^b^	5.1% ^b^	4.3% ^a^	11.8% ^b^
**In general, if you get the flu, how much impact do you think having the flu would affect your daily life?**							
A great deal	32.8%	22.5% ^a^	33.3% ^b^	38.8% ^b^	35.0% ^b^	43.5% ^a^	25.0% ^b^
Somewhat	37.7%	37.3%	37.3%	34.6%	42.6%	35.3%	39.2%
A little	25.4%	33.5% ^a^	24.5% ^b^	24.3% ^b^	19.8% ^b^	17.8% ^a^	31.0% ^b^
Not at all	4.1%	6.7% ^a^	4.9% ^a,b^	2.3% ^b^	2.5% ^b^	3.4%	4.8%
**In general, if you get the flu, how likely do you think it is you could pass the flu on to someone else?**							
Very likely	31.8%	25.1% ^a^	33.8% ^b^	32.2% ^a,b^	34.5% ^b^	42.7% ^a^	24.3% ^b^
Likely	41.7%	49.8% ^a^	43.7% ^a,b^	39.0% ^b,c^	34.0%^c^	38.3%	43.7%
Unlikely	20.4%	18.5%	18.5%	23.1%	22.3%	13.0% ^a^	25.7% ^b^
Very unlikely	6.1%	6.8% ^a,b^	4.0% ^b^	5.7% ^a,b^	9.1% ^a^	6.0%	6.2%
**Self-reported influenza experience**							
**Have you ever been sick with influenza or flu—that is, flu which infected your lungs?**							
Yes	42.4%	39.1%	42.6%	42.4%	44.1%	47.1% ^a^	38.9% ^b^
No	57.8%	60.9%	57.4%	57.6%	55.9%	52.9% ^a^	61.1% ^b^
**How would you rate your last influenza or flu illness? (‘1’—not very severe to ‘5’—very severe)**							
‘1’ or ‘2’	35.0%	43.9% ^a^	29.8% ^b^	26.2% ^b^	47.6% ^a^	32.4%	37.4%
‘3′	36.1%	39.4% ^a,b^	42.7% ^b^	33.0% ^a,b^	26.8% ^a^	36.9%	34.8%
‘4’ or ‘5’	28.8%	16.7% ^a^	27.5% ^a^	40.8% ^b^	25.6% ^a^	30.7%	27.8%
**How many times would you estimate you have had influenza- or the flu—in your life?**							
Never	21.4%	26.6% ^a^	18.3% ^b^	21.1% ^a,b^	21.8% ^a,b^	19.0%	23.2%
1–2 times	31.7%	41.0% ^a^	27.6% ^b^	28.0% ^b^	33.5% ^a,b^	32.7%	30.9%
3–4 times	27.3%	23.4%	30.0%	29.3%	23.9%	25.5%	28.6%
5–10 times	13.9%	7.4% ^a^	17.9% ^b^	15.0% ^b^	12.8% ^a,b^	14.8%	13.2%
11 or more times	5.7%	1.6% ^a^	6.2% ^b^	6.5% ^b^	8.0% ^b^	8.1% ^a^	4.1% ^b^

Note: Comparisons are made across columns by row using the Bonferroni adjusted *p*-value approach with an alpha value of ≤0.05. All possible pairwise comparisons are made between categories of a given variable (e.g., age). For any given variable, rows with no superscripts have no significant differences. Within rows that do have superscripts, any two values that share a superscript are not significantly different from one another; values that do not have a common superscript are significantly different (e.g., a, b, and c are significantly different from one another). For example, in the table there are no significant differences based on age in the percentage of respondents who reported being “not at all informed” about the seasonal flu vaccine, but there are significant differences based on age for all other categories of that same variable.

**Table 5:** Beliefs regarding influenza vaccine.

	Overall	Age				Got Flu Vaccine in Last 12 Months	
		19–30	31–49	50–64	65+	Yes	No
*N*	*1005*	*212*	*330*	*264*	*199*	*418*	*571*
**How well informed are you about who should receive the seasonal flu vaccine?**							
Very well informed	53.0%	36.9% ^a^	54.9% ^b^	53.3% ^b^	64.6% ^c^	69.3% ^a^	40.9% ^b^
Somewhat informed	37.2%	44.4% ^a^	36.1% ^a,b^	38.2% ^a,b^	30.8% ^b^	28.7% ^a^	43.4% ^b^
Not well informed	6.6%	12.8% ^a^	5.3% ^b,c^	6.9% ^c^	2.6% ^b^	1.0% ^a^	10.7% ^b^
Not at all informed	3.2%	5.9% ^a^	3.8% ^a,b^	1.5% ^b^	2.1% ^a,b^	1.0% ^a^	5.0% ^b^
**How well informed are you about the benefits associated with getting seasonal flu vaccine?**							
Very well informed	50.7%	37.3% ^a^	48.0% ^b^	52.3% ^b^	65.7% ^c^	69.0% ^a^	37.2% ^b^
Somewhat informed	38.2%	42.2% ^a^	43.9% ^a^	35.9% ^a,b^	28.8% ^b^	27.8% ^a^	45.8% ^b^
Not well informed	6.8%	14.6% ^a^	3.4% ^b^	7.4% ^c^	3.5% ^b,c^	2.2% ^a^	10.0% ^b^
Not at all informed	4.3%	5.9% ^a^	4.7% ^a,b^	4.3% ^a,b^	2.0% ^b^	1.0% ^a^	7.0% ^b^
**How well informed are you about the risks associated with not getting a seasonal flu vaccine?**							
Very well informed	49.6%	34.8% ^a^	49.1% ^b^	49.0% ^b^	65.5% ^c^	66.7% ^a^	37.3% ^b^
Somewhat informed	36.9%	39.6% ^a^	39.7% ^a^	36.9% ^a,b^	29.9% ^b^	27.7% ^a^	43.4% ^b^
Not well informed	9.2%	17.6% ^a^	7.8% ^b^	9.8% ^b^	2.5% ^c^	4.4% ^a^	12.7% ^b^
Not at all informed	4.3%	8.0% ^a^	3.4% ^b^	4.3% ^a,b^	2.0% ^b^	1.2% ^a^	6.6% ^b^
**How easy or difficult is it—or would it be—for you to get a flu shot if you wanted one?**							
Very easy	80.5%	64.3% ^a^	79.4% ^b^	85.5% ^b,c^	91.0% ^c^	90.3% ^a^	73.1% ^b^
Somewhat easy	14.5%	24.7% ^a^	14.1% ^b^	12.6% ^b,c^	8.0% ^c^	8.2% ^a^	19.5% ^b^
Somewhat difficult	3.1%	9.3% ^a^	2.6% ^b^	1.5% ^b,c^	0.0% ^c^	0.5% ^a^	5.0% ^b^
Very difficult	1.9%	1.6% ^a,b^	3.9% ^b^	0.4% ^a^	1.0% ^a,b^	1.0%	2.4%
**How confident are you in the safety of the seasonal flu vaccine or shot? (‘1’—not very confident to ‘5’—very confident)**							
‘1’ or ‘2’	27.8%	34.3% ^a^	35.4% ^a^	20.1% ^b^	18.7% ^b^	7.9% ^a^	42.5% ^b^
‘3’	22.9%	30.0% ^a^	25.0% ^a^	23.1% ^a^	11.6% ^b^	15.3% ^a^	28.2% ^b^
‘4’ or ‘5’	49.3%	35.7% ^a^	39.6% ^a^	56.8% ^b^	69.7% ^c^	76.7% ^a^	29.3% ^b^
**How confident are you in the effectiveness of the seasonal flu vaccine or shot?**							
‘1’ or ‘2’	30.7%	33.0% ^a,b^	40.9% ^b^	25.1% ^a,c^	19.0% ^c^	9.9% ^a^	46.3% ^b^
‘3’	28.7%	40.2% ^a^	29.9% ^b^	28.5% ^b^	14.9% ^c^	21.0% ^a^	34.1% ^b^
‘4’ or ‘5’	40.6%	26.8% ^a^	29.3% ^a^	46.4% ^b^	66.2% ^c^	69.1% ^a^	19.6% ^b^
**How confident are you that you would benefit from receiving a seasonal flu vaccine or shot?**							
‘1’ or ‘2’	36.0%	42.7% ^a^	45.9% ^a^	29.4% ^b^	21.4% ^b^	10.1% ^a^	55.5% ^b^
‘3’	21.1%	29.9% ^a^	21.1% ^b^	22.5% ^a,b^	9.7% ^c^	14.4% ^a^	25.0% ^b^
‘4’ or ‘5’	43.0%	27.5% ^a^	33.0% ^a^	48.1% ^b^	68.9% ^c^	75.5% ^a^	19.5% ^b^

Note: Comparisons are made across columns by row using the Bonferroni adjusted *p*-value approach with an alpha value of ≤0.05. All possible pairwise comparisons are made between categories of a given variable (e.g., age). For any given variable, rows with no superscripts have no significant differences. Within rows that do have superscripts, any two values that share a superscript are not significantly different from one another; values that do not have a common superscript are significantly different (e.g., a, b, and c are significantly different from one another). For example, in the final row of results, only 27.5% of 19–30 year olds reported a ‘4’ or ‘5’ on the measure of confidence in the benefit of receiving a flu vaccine. This percentage is not significantly different than the percentage of 31–49 year olds (33.0%), but is significantly lower than the percentage of 50–64 year olds (48.1%) and 65+ year olds (68.9%). The 65+ group had a significantly higher percentage report either a ‘4’ or ‘5’ than all other age groups, as indicated by their unique superscript.

**Table 6:** Regressions predicting (a) likelihood of getting an influenza vaccine during the upcoming (2016–2017) influenza season, (b) number of years vaccinated for influenza for the three years 2013, 2014, and 2015, and (c) Influenza vaccine confidence.

	Flu Shot Likelihood (2016–2017)	Flu Shot Receipt (2013–2015)	Flu Shot Confidence
***Block 1: Demographics***			
Gender (Female coded high)	−0.05 *	−0.04	−0.02
Age	0.10 ***	0.13 ***	0.12 ***
Race/ethnicity (Black)	0.06 **	0.03	0.06
Race/ethnicity (Hispanic)	−0.07 **	−0.06 *	0.08 *
Race/ethnicity (Other)	0.03	0.02	−0.05
Household income	0.00	0.04	−0.09 **
Education	0.00	−0.05	0.05
***Block 2: Awareness***			
Informed about flu vaccine	0.03	0.07 *	0.18 ***
Aware of flu vaccine recommendation	0.14 ***	0.16 ***	0.26 ***
***Block 3: Physician advice***			
Physician flu vaccine recommendation	0.17 ***	0.19 ***	0.09 **
***Block 4: Vaccine Status***			
Perceived likelihood of getting the flu	0.13 ***	0.19 ***	0.27 ***
Flu seriousness	−0.01	−0.04	0.07
Flu consequences	−0.02	0.00	0.03
Flu contagiousness	0.05	0.05	0.03
***Block 5: Confidence***			
Confidence in flu vaccine	0.51 ***	0.38 ***	n.a.
***Block 6: Barriers***			
Difficulty of getting flu shot	0.02	0.04	0.06 *
Total R^2^ (%)	63.1	56.9	41.2

Notes: (1) * *p* ≤ 0.05, ** *p* ≤ 0.01, *** *p* ≤ 0.001; (2) Cell entries for all blocks are standardized regression coefficients.

**Table 7:** Logistic regression predicting whether respondents received a flu shot in the last 12 months where the reference group is “Yes” (they plan to have their child or children get all the remaining recommended vaccines).

	Predicted Likelihood B (S.E.)
	Yes
Intercept	−9.09 (1.18) ***
Gender (Female)	0.44 (0.23)
Age	0.18 (0.07) **
Race (Black)	0.59 (0.37)
Race (Hispanic)	−1.26 (0.39) **
Race (Other)	0.70 (0.38)
Household Income	0.06 (0.03) *
Education	0.01 (0.06)
Informed about flu vaccine	0.35 (0.20)
Aware of flu vaccine recommendation	1.64 (0.36) ***
Physician recommendation	1.63 (0.24) ***
Perceived likelihood of getting the flu	0.50 (0.13) ***
Flu seriousness	0.08 (0.18)
Flu consequences	−0.22 (0.16)
Flu contagiousness	0.02 (0.14)
Confidence in flu vaccine	0.90 (0.11) ***
Difficulty of getting flu shot	0.00 (0.20)
-2 Log likelihood	555.14
Nagelkerke R^2^	0.64
N	774

*Notes*: (1) * *p* ≤ 0.05, **p ≤ 0.01, ***p ≤ 0.001; (2) Nagelkerke index is adjusted so that the maximum value it can attain is 1.00 [27].
